# Plasma Kynurenic Acid Concentration in Patients Undergoing Cardiac Surgery: Effect of Anaesthesia

**DOI:** 10.1007/s00005-014-0312-z

**Published:** 2014-09-10

**Authors:** Edyta Kotlinska-Hasiec, Patrycja Nowicka-Stazka, Jolanta Parada-Turska, Krzysztof Stazka, Janusz Stazka, Przemyslaw Zadora, Wojciech Dabrowski

**Affiliations:** 1 Department of Anaesthesiology and Intensive Therapy, Medical University of Lublin, Jaczewskiego 8, 20-954 Lublin, Poland; 2 Department of Endocrinology, Medical University of Lublin, Lublin, Poland; 3 Department of Rheumatology and Connective Tissue Diseases, Medical University of Lublin, Lublin, Poland; 4 Department of Cardiology, Medical University of Lublin, Lublin, Poland; 5 Department of Cardiac Surgery, Medical University of Lublin, Lublin, Poland

**Keywords:** Kynurenic acid, Neutrophil/lymphocyte ratio, Sevoflurane, Cardiac surgery, General anaesthesia

## Abstract

Increases in plasma kynurenic acid (KYNA) concentration relate to the severity of inflammation. The aim of this study was to analyse changes in plasma KYNA concentration and neutrophil/lymphocyte ratio (NLR) in cardiac surgery patients. Additionally, the effect of anaesthesia was analysed. Adult cardiac surgery patients under intravenous general anaesthesia were studied. Additionally, some patients received sevoflurane (SEV) prior to cardiopulmonary bypass. Plasma KYNA concentration and NLR were measured before anaesthesia, just after surgery and on postoperative days 1, 2 and 3. Patients were assigned to two groups: patients who did not receive SEV (NonSEV group) and patients who received SEV (SEV group). Forty-three patients were studied. Twenty-four of them received SEV. KYNA increased immediately after surgery and remained elevated through postoperative day 3 in the NonSEV group, whereas it was similar to the preoperative concentration in the SEV group. NLR increased immediately after surgery in both groups, and higher values were noted in the NonSEV group than in the SEV group at postoperative days 2 and 3. Plasma KYNA concentration correlated with NLR in the NonSEV group. Cardiac surgery caused an increase in NLR. Plasma KYNA increased in the NonSEV group and correlated with NLR. Administration of SEV inhibited the increase in KYNA, most likely due to its anti-inflammatory properties.

## Introduction

**Fig. 1:**
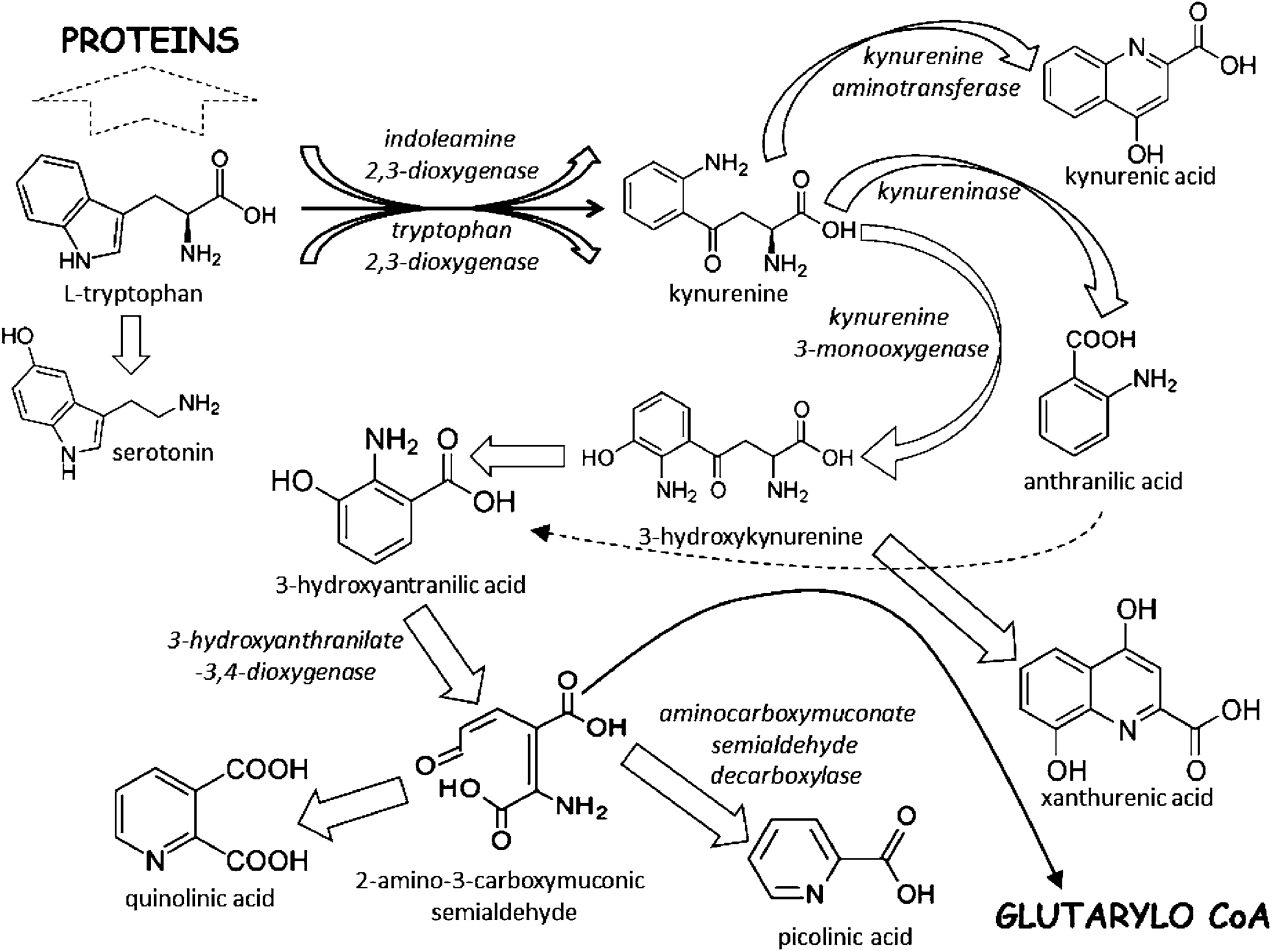
Kynurenine pathway

Kynurenic acid (KYNA) is one of the end products of tryptophan formed in the kynurenine pathway. In the first step of this process, tryptophan is oxygenised by tryptophan 2,3 dioxygenase (TDO) or indoleamine 2,3-dioxygenase (IDO) into kynurenine, which is then transformed by kynurenine aminotransferases into KYNA (Fig. 1). Physiological concentration of the human plasma KYNA ranges between 25 and 60 nM (Bender 1982; Forrest et al. 2006; Turski et al. 2013). Several pathologies affect plasma KYNA concentration. It becomes elevated following brain ischaemia and stroke or in schizophrenia, whereas it decreases during Alzheimer’s disease, multiple sclerosis, epilepsy or depression (Angus et al. 2001; Darlington et al. 2007; Hartai et al. 2007; Laupland et al. 2004; Némath et al. 2005; Nilsson et al. 2005; Stone et al. 2003). Moreover, a lot of studies underline the neuroprotective properties of KYNA in animals with experimental brain injury (Darlington et al. 2007; Gellért et al. 2013; Sas et al. 2008). KYNA production is also elevated during a bacterial or viral inflammation, an autoimmune disease and after a severe trauma (Forrest et al. 2006; Hartai et al. 2007; Scott et al. 2009; Zeden et al. 2010). Increases in plasma tryptophan metabolites have also been observed in patients undergoing elective abdominal or cardiac surgery (Forrest et al. 2011; Marfella et al. 1999). Perioperative increases in plasma cytokine concentrations following surgery-related inflammatory response may affect plasma KYNA concentration. Interestingly, the severity of surgery-related inflammatory response appears to depend on perioperative treatment and on type of anaesthesia. Some authors documented a significant reduction in plasma levels of pro-inflammatory cytokines following ketamine or propofol administration (Blum and Zuo 2013; Gokcinar et al. 2013). Similarly, volatile anaesthetics present anti-inflammatory effect reducing pro-inflammatory cytokine production in the kidney, respiratory system and nervous system (Li et al. 2013a, b; Kim et al. 2011). Moreover, the inhalation of sevoflurane (SEV) at 1.0 minimal anaesthetic concentration (MAC) significantly attenuates concentrations of pro-inflammatory cytokines such as tumor necrosis factor (TNF)-α and interleukin (IL)-1β in plasma of septic shock rats (Hofstetter et al. 2007). Based on these observations, we can assume that SEV anaesthesia may affect plasma KYNA concentration via an attenuation of a surgery-related inflammatory response, but this effect has not been examined yet.

The neutrophil/lymphocyte ratio (NLR) is a potentially interesting marker of inflammation. A greater perioperative neutrophil count may reflect subclinical inflammation, and lymphocyte count relates to physiological stress and is a surrogate marker for general health (de Jager et al. 2010; Zahorec 2001). Therefore, NLR can be proposed as a simple marker of inflammation. Many authors describe NLR as a marker of postoperative outcome (Azab et al. 2013; Gibson et al. 2007, 2010). Both an elevated neutrophil count and a reduced lymphocyte count are associated with a worse outcome after cardiac surgery (Azab et al. 2013; Gibson et al. 2007). Elevated NLR is associated with an increased risk of postoperative atrial fibrillation, which may correspond to a greater severity of postoperative inflammation (Gibson et al. 2010). Based on this observation, we hypothesised that NLR correlates with plasma KYNA concentration.

The aim of the present study was to analyse the changes in plasma KYNA concentration and in NLR in patients undergoing cardiac surgery with cardiopulmonary bypass (CPB). Additionally, the effects of SEV, a pleasant-smelling volatile anaesthetic, widely used for induction and maintenance of general anaesthesia, on plasma KYNA concentration and NLR were analysed.

## Patients and Methods

The study was approved by the Committee for Bioethics at the Medical University of Lublin, and written informed consent was obtained from all patients involved. Patients scheduled for elective cardiac surgery due to stable angina pectoris (CABG) or aortic/mitral valve in sufficiency were included. The exclusion criteria encompassed the following: any current neurological disease or history of neurological disorders, brain surgery, severe head trauma, significant carotid artery stenosis, chronic respiratory disease, chronic renal insufficiency, chronic renal failure or an EuroScore higher than 8.

### Anaesthesia and Surgery

One day before surgery, all participants received the same premedication with lorazepam (Lorafen, Polfa, Inc., Poland) and morphine hydrochloride (morphinum hydrochloricum, Polfa, Inc., Poland). Anaesthesia was induced using fentanyl (Fentanyl, Polfa, Poland) midazolam (Sopodorm, Polfa, Inc., Poland) and etomidate (Etomidate, Braun, Germany). Muscle relaxation was obtained with a single dose of pancuronium (Pavulon, Pancuronium, Jelfa, Poland). After tracheal intubation, all patients were ventilated using intermittent positive pressure ventilation with a mixture of air and oxygen. Parameters were adjusted to maintain normocapnia, which was controlled by blood gas analysis. Anaesthesia was maintained throughout the procedure using remifentanil (Ultiva, GlaxoSmithKline, Plc., UK) and propofol (Diprivan, Astra-Zeneca, Ltd., USA). Additionally, prior to initiating CPB, some patients received SEV (Sevorane, Abbott, Plc., UK) at a dose of 0.5–1.0 of the MAC. The concealed envelope method was used for SEV administration. The dose of anaesthetic administered depended on the patient’s haemodynamic status. Intra-operative hypertension was treated with a single bolus of remifentanil or propofol. In patients not responding adequately to anaesthesia, a single intravenous dose of urapidil (Ebrantil, Takeda GmbH, Germany) was used. Tachycardia was treated with beta-blockers.

Prior to CPB, heparinum sulphuricum (Heparin, Polfa, Poland) was administered, and the activated clotting time was controlled up to 400 s. For CPB, standard cannulation of the ascending aorta and inferior vena cava was performed through the right atrium. During CPB, circulation and ventilation were maintained with the heart–lung machine S III (Stöckert, GmbH, Germany), and the mean arterial pressure was kept between 45 and 105 mmHg. In all patients, the volume of priming was constant and consisted of 1,000 ml of Ringer’s solution (Ringer, Baxter, Inc., Sabinanico, Spain), 500 ml of 6 % solution of hydroxyethylated starch (Voluven, Fresenius-Kabi, Inc., Poland), 250 ml of 20 % mannitol (Mannitol, Fresenius-Kabi, Poland), 20 ml of sodium hydroxycarbonate (natrium bicarbonatum, Polfarma, Inc., Poland) and 75 mg of heparinum sulphuricum. Cardiopulmonary bypass was instituted with pulsatile flow of 2.4 l/min/m^2^ of body surface area. After traditional aortic clamping, myocardial viability was preserved with antegrade hyperkalaemic warm blood cardioplegia. During mild hypothermic CPB, the mean arterial pressure, haematocrit and blood gas parameters as well as the lactate, sodium and potassium levels were measured. Distal anastomoses were performed during cardioplegic arrest, whereas proximal anastomoses were performed with resumed perfusion and a side-biting clamp. Mediastinal blood was sucked into the cardiotomy reservoir of the heart–lung machine. The last suction was performed 10 min before the completion of CPB. In all cases, separation from the heart–lung machine was uneventful, and intra-aortic counterpulsation was not necessary. In patients requiring inotropic support, dobutamine (Dobuject, Bayer-Schering, Gmbh, Germany) or dobutamine and norepinephrine (Levonor, Polfa, Poland) infusion were used at doses that depended on the patient’s haemodynamic status. The effect of heparin was reversed by an adequate dose of protamine sulphate (Protaminum sulphuricum, Biomed, Inc., Poland). During surgery and in the early postoperative period, patients received an infusion of mixture of potassium chloride and magnesium sulphate.

After surgery, patients were sent to the postoperative intensive care unit (PICU). All of them were ventilated using synchronised intermittent mandatory ventilation with pressure support. Patients were extubated 8–12 h after surgery and were transferred from the PICU on the second or third postoperative day.

After the induction of anaesthesia and prior to the beginning of CPB, 500 ml of 6 % hydroxyethyl starch (6 % HAES, Polfa, Poland) was infused. After CPB, haematologic parameters were monitored. None of the patients required massive fluid resuscitation, and the type and dose of administered fluids depended upon the patient’s haemodynamic status. Intravascular fluid insufficiency during the early postoperative period was treated by supplementation with gelatine preparations or electrolyte fluids (PWE and Ringer, Polfa, Poland).

### Study Protocol and Patient Distribution

Plasma KYNA concentration and NLR were measured at five time points: before anaesthesia and surgery, just after completion of surgery and on the morning of postoperative days 1, 2 and 3. The blood samples for plasma KYNA concentration measurement were collected from the radial artery and immediately centrifuged (2,500 r/min); obtained plasma was frozen at −20 °C. Plasma KYNA was measured fluorometrically. Plasma was deproteinated with 50 % trichloroacetic acid and centrifuged. Supernatant was applied on cation-exchange resin (Dowex 50 W+, Sigma-Aldrich, MO, USA). Eluted KYNA was subjected to high-performance liquid chromatography (HPLC) (Hewlett Packard 1050 HPLC system: ESA catecholamine HR-30, 3 μm, C_18_ reverse-phase column) and quantified fluorometrically (Hewlett Packard 1046A fluorescence detector: excitation 344 nm, emission 398 nm) (Shibata 1988). The KYNA concentrations are expressed in nM.

Based on the volatile anaesthetic administration, patients were assigned to two groups: patients who did not receive SEV (NonSEV group) and patients who received SEV before the beginning of CPB (SEV group).

### Statistics

Means and standard deviations (SD) were calculated for parametric data. The value at time point 1 was regarded as baseline. The unpaired Student’s *t* test was used to analyse variables with a normal distribution. Non-parametric data were statistically analysed using the Wilcoxon signed-rank test and the Kruskal–Wallis ANOVA test for initial detection of differences. *p* < 0.05 was considered statistically significant. Sample size was determined by Statistica 9 software. The power of all statistical tests was determined by G*Power software (1–β).

## Results

Forty-three consecutive patients (30 male and 13 female) were examined. In all cases, weaning from the heart–lung machine was uneventful and intra-aortal counterpulsation was not necessary. After CPB, none of the patients required an aggressive fluid therapy.

The mean age of all patients was 63 ± 12 years. It was 63 ± 13 years in patients who did not receive SEV and 62 ± 12 years in patients who received SEV before CPB. There was no significant difference in body mass index between both studied groups (Table 1). Moreover, there was no difference in mean duration of anaesthesia, surgery, CPB or aorta clamping (Table 1). Twenty-four patients (55.8 %) received SEV before the CPB.

**Table 1:** Patient demographic data

	Study population	NonSEV group (*n* = 19)	SEV group (*n* = 24)	*p* value
Male	30	12	18	–
Female	13	7	6	–
BMI (kg/m^2^)	28.44 ± 5.53	28.50 ± 5.42	28.37 ± 5.83	0.91
Duration of (min)				
Anaesthesia	282 ± 68	291 ± 79	275 ± 60	0.59
Surgery	222 ± 65	235 ± 74	212 ± 60	0.23
CPB	112 ± 59	124 ± 74	103 ± 45	0.54
AC	70 ± 39	74 ± 44	66 ± 36	0.56

*BMI* body mass index, *AC* aorta clamping, *CPB* cardiopulmonary bypass, *SEV* sevoflurane. *NonSEV group* patients who were anesthetised intravenously, *SEV group* patients who were anesthetised intravenously and received SEV inhalation before CPB

**Fig. 2:**
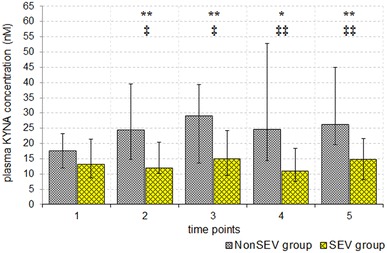
Changes in plasma KYNA concentration [median (quartile 1 and 3)] in patients who were anaesthetised intravenously and did not receive volatile anaesthetic (sevoflurane) prior to cardiopulmonary bypass (CPB) (NonSEV group) and patients who were anaesthetised intravenously and received sevoflurane (SEV group). Time points: *1* before anaesthesia and surgery (baseline); *2* just after surgery; *3* on the morning of postoperative day 1; *4* on the morning of postoperative day 2; and *5* on the morning of postoperative day 3. *p < 0.05, **p < 0.01 compared with baseline in the NonSEV group (Wilcoxon test). ^**‡**^ *p* < 0.05, ^**‡‡**^ *p* < 0.01, NonSEV group vs. SEV group

Before anaesthesia surgery, there was no statistically significant difference in plasma KYNA concentrations between NonSEV and SEV groups [median (quartile 1; quartile 3): 17.51 nM (11.91; 23.25) in the NonSEV group vs. 13.24 nM (8.73; 21.27) in the SEV group]. In the NonSEV group, increased KYNA was detected in plasma obtained immediately after completion of the surgical procedure and 1, 2 and 3 days thereafter. In the SEV group, plasma KYNA concentration was unaffected during postoperative period (Fig. 2). Moreover, plasma KYNA was significantly lower in the SEV group than in the NonSEV group immediately after surgery and on the postoperative days 1, 2 and 3 (Fig. 2).

**Fig. 3:**
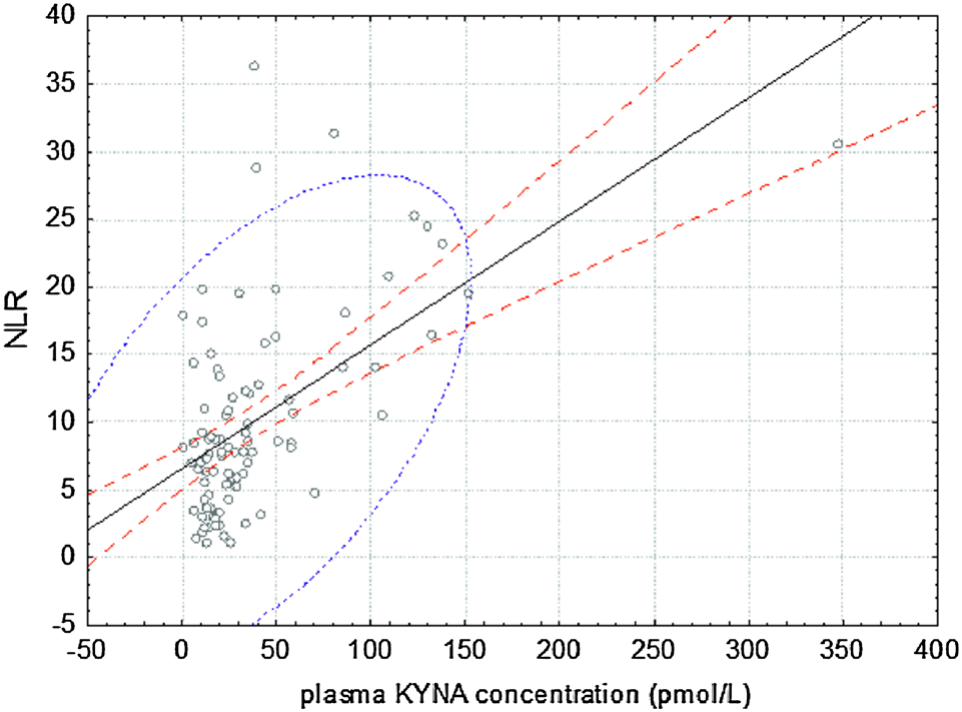
The overall correlation between plasma KYNA concentration and neutrophil/lymphocyte ratio (NRL) in patients who were anesthetised intravenously (NonSEV group) (Spearman correlation test). In the SEV group, plasma KYNA concentration did not correlate with NLR at consecutive time points, and there was no overall correlation between plasma KYNA concentration and NLR

The value of NLR increased immediately after surgery in both groups, and significantly higher values were noted on the morning of postoperative days 2 and 3 (Table 2). There was an overall correlation between plasma KYNA concentration and NLR in the NonSEV group (*p* < 0.001, *r* = 0.46; Fig. 3). Moreover, plasma KYNA concentration strongly correlated with NLR on the postoperative days 1 and 2 (*p* < 0.01, *r* = 0.56 and *p* < 0.01, *r* = 0.5, respectively) and moderately on the postoperative day 3 in the NonSEV group (*p* < 0.05, *r* = 0.47).

**Table 2:** The analysis of changes in neutrophil/lymphocyte ratio in cardiac surgery patients only anaesthetised intravenously (NonSEV group) and patients who were anaesthetised intravenously and received sevoflurane (SEV) at a dose of 0.5–1.0 of the minimal anaesthetic concentration, prior to initiating cardiopulmonary bypass (SEV group)

Patients	Parameter	Time point				
		1	2	3	4	5
NonSEV group	Median (quartile 1 and 3)	3.12 (2.47–4.5)	10.8* (7.07–15.15)	9.9* (6.7–14.61)	10.52* (6.32–13.73)	8.13* (6.38–14.4)
SEV group	Median (quartile 1 and 3)	2.49 (2–3.13)	11.78* (6.26–15.21)	10.56* (7.57–13.75)	7.6* (6.71–9.01)	6.85* (5.47–8.08)
Intergroup differences (*p*)		0.33	0.52	0.52	0.001	0.02

Time points: *1* before anaesthesia and surgery (baseline), *2* just after surgery, *3* on the morning of postoperative day 1, *4* on the morning of postoperative day 2, and *5* on the morning of postoperative day 3

* *p* < 0.001 compared with baseline in the NonSEV group (Wilcoxon test)

## Discussion

This is the first study documenting the effect of SEV anaesthesia on plasma KYNA concentration in patients undergoing elective cardiac surgery with CPB and its correlation with NLR. We found that cardiac surgery resulted in an increase in plasma KYNA in patients anaesthetised intravenously. An increase in KYNA was recorded in blood obtained immediately after completion of the surgical procedure and persisted until day 3 of observation. In these patients, plasma KYNA concentration correlated with NLR. In contrast, plasma KYNA concentration remained unaltered and did not correlate with NLR in patients who were anaesthetised intravenously and received SEV before the beginning of CPB.

Perioperative measurement of plasma KYNA concentration may provide a large amount of information and predispose final outcome. Persisted high plasma KYNA concentration has been noted in non-survived stroke traumatic patients and patients, who died after resuscitation (Darlington et al. 2007; Ploder et al. 2009; Ristagno et al. 2013). Significantly higher plasma KYNA concentration has been found in carotid surgery patients with postoperative neurological dysfunctions (Terlecki et al. 2014). KYNA has been also suggested as a marker of distress intolerance (Chiapelli et al. 2014). Noteworthy, increasing plasma KYNA concentration might predispose to sepsis and septic shock in patients after multi trauma and its level is related to sseverity of infection (Dabrowski et al. 2014; Schefold et al. 2009; Zeden et al. 2010). All of the above-mentioned pathologies may complicate early postoperative period in cardiac surgery patients.

The effect of cardiac surgery on plasma KYNA concentration has been poorly documented. Forrest et al. (2011) reported no difference in blood KYNA in patients undergoing CABG during and up to 2 days after cardiac surgery and an increase in plasma KYNA on the sixth postoperative day. They also noted no changes in plasma KYNA in patients undergoing thoracic surgery. Importantly, all of the CABG patients were anaesthetised intravenously, and 71.4 % of them received volatile anaesthetics. All patients undergoing thoracic surgery were anaesthetised intravenously, and 50 % of them received isoflurane (Forrest et al. 2011). It is noteworthy that the effect of anaesthesia on plasma KYNA was not analysed in that study.

Our finding is in good agreement with that of Forrest et al. (2011). In our study, plasma KYNA did not change in patients who received a volatile anaesthetic, SEV, before the beginning of CPB. In contrast to Forrest et al. (2011), we found that cardiac surgery increased the KYNA concentration in patients anaesthetised intravenously and that KYNA was significantly higher than in patients who received SEV. Therefore, we can infer that cardiac surgery increases plasma KYNA and the use of volatile anaesthetic blocks this effect.

An increase in plasma KYNA recorded in patients anaesthetised intravenously may result from surgery-induced inflammatory response. Raised blood KYNA has been found in septic patients (Dabrowski et al. 2014; Schefold et al. 2009; Zeden et al. 2010), HIV-1 infection (Heyes et al. 1992), chronic kidney disease (Schefold et al. 2009), stroke patients (Darlington et al. 2007), after severe brain injury (Mackay et al. 2006) and cardiac arrest (Ristagno et al. 2013). Moreover, increased salivary KYNA concentration has been observed after acute physiological stress (Chiapelli et al. 2014). KYNA is regarded as an end-product of one of the branches of the kynurenine pathway, which is the main pathway of tryptophan metabolism. More than 90 % of plasma tryptophan is metabolised via the kynurenine pathway (Bender 1982). This process strongly depends on IDO and/or TDO activity. TDO has been found primarily in the liver and is activated by high tryptophan concentration and corticosteroids (Bender 1982; Braidy et al. 2011; Ruddick et al. 2006; Turski et al. 2013). IDO has been identified in brain and liver as well as in monocytes, macrophages, fibroblasts, dendritic cells and microglia. It is activated by several inflammatory mediators, such as TNF-α, IL-1, IL-2, IL-6, IL-23, interferon (IFN)-α and IFN-γ. IFN-γ is considered the most powerful activating factor (Bender 1982; Boasso et al. 2008; Braidy et al. 2011; Kidani et al. 2005; Song et al. 2013; Turski et al. 2013; Zitta et al. 2010). IDO activity stimulated by cytokines leads to a substantial increase in the level of tryptophan catabolites, including KYNA. Interestingly, plasma TNF-α, IL-1β, IL-6 and IL-10 and IFN-γ increase following CPB (Codaccioni et al. 2009; Thal et al. 2012; Wang et al. 2010). Additionally, some authors have noted similarities between endotoxin-induced immune defect and cardiac surgery (Codaccioni et al. 2009). This inflammatory response is initiated by surgical trauma, blood contact with artificial surfaces in CPB circuit and ischemia–reperfusion injury, and the degree of inflammatory response corresponds to postoperative outcome, including neuropsychological disorders (Symons and Myles 2006; Yue et al. 2008). In the present study, we observed an overall correlation between plasma KYNA concentration and NLR, a marker of inflammation (de Jager et al. 2010; Gibson et al. 2010; Zahorec 2001), in the NonSEV group. Moreover, we noted a strong correlation between plasma KYNA concentration and NLR in the early postoperative period, when the inflammatory response was the most intense. Therefore, we can assume that the postoperative increase in plasma KYNA is a result of CPB-related inflammatory response, though further studies are required to support our findings and this hypothesis.

An inflammation-caused increase in plasma KYNA may be confirmed by its correlation with NLR in patients anaesthetised intravenously. Lymphocytopenia and neutrophilia are physiological responses to inflammation in several pathologies. This so-called neutrophil–lymphocyte stress factor is a well-documented marker describing the severity of disease and outcome measured by Acute Physiology and Chronic Health Evaluation II and Sepsis-related Organ Failure Assessment scores (Hwang et al. 2012; Zahorec 2001). Moreover, NLR is considered an independent predictor of bacteraemia and severity of inflammation in several pathologies (de Jager et al. 2010; Shiny et al. 2014). NLR often increases following cardiac surgery (Azab et al. 2013; Gibson et al. 2007, 2010). NLR is also a sensitive marker of inflammation predicting postoperative atrial fibrillation in cardiac surgery patients. Interestingly, Brouns et al. (2010) found a correlation between tryptophan oxidation and NLR in stroke patients. They concluded that tryptophan metabolism strictly correlated with the stroke-related inflammatory response measured by C-reactive protein, erythrocyte sedimentation rate and NLR. They did not analyse a relationship between KYNA and NLR ratio, but they presented a similar trend of changes in plasma KYNA concentration and NLR. A relation between NLR and plasma KYNA concentration was observed by Terlecki et al. (2014), who analysed changes in plasma KYNA concentration and NLR and found a strong correlation between mentioned parameters. In the present study, we also found a correlation between NLR and plasma KYNA concentration in patients anaesthetised intravenously. Based on these findings, we can propose KYNA as a marker of inflammation.

Several volatile anaesthetics may exert anti-inflammatory effects when administered before or just after injury. They affect the interaction between neutrophils and endothelium, reducing the levels of TNF-α and IL-6 in human endothelial cells (Forrest et al. 2010; Homi et al. 2010; van Harten et al. 2012; Yue et al. 2008). Some experimental studies have shown a significant reduction of inflammatory mediators, chemotaxis and neutrophil adherence in a model of acute lung injury (van Harten et al. 2012). Inhalation of SEV decreases TNF-α, IL-6 and IL-8 in human cells and in rat lung (Rodríuez-González et al. 2013; Song et al. 2013; Watanabe et al. 2013; Yue et al. 2008). Moreover, inhalation of SEV significantly reduces IFN-γ production in mice (Polak et al. 2012). Similarly, isoflurane inhibits the aforementioned proinflammatory cytokine production in lung tissue and suppresses apoptosis by downregulating procaspases 3 and 8 and caspases 3 and 8 (Li et al. 2013b). Notably, both SEV and isoflurane ameliorate inflammation, but SEV is significantly more inhibitory than isoflurane in this respect (Bedirli et al. 2012).

It seems that the elevation in plasma KYNA may also result from the severity of CPB-related cardiac dysfunction. KYNA content correlates with severity of cardiac failure (Ristagno et al. 2013). In the present study, plasma KYNA increased in patients who were anaesthetised intravenously and were not subjected to SEV. It started immediately after the surgery and persisted for 72 postoperative hours. Similarly, the postoperative myocardial stunning, which occurs in more than 90 % of patients undergoing CPB, persists for 72 postoperative hours and clinically manifests by low cardiac output, frequently requiring pharmacologic or mechanical support (Domanski et al. 2011; Kinoshita and Asai 2012; Malik et al. 2006). There is also evidence that the use of SEV markedly reduces the postoperative cardiac dysfunction and improves its haemodynamic function (Lango and Mroziński 2010; Soro et al. 2012). Likewise, in our patients receiving SEV, no changes in plasma levels of KYNA were observed. Thus, we can speculate that SEV-related improvement in cardiac function affects plasma KYNA concentration, but this potential relationship should be confirmed in the future.

It is noteworthy that volatile anaesthetics reduce ischemic brain damage and improve the neurologic outcome (Brosnan and Thiesen 2012; Ding et al. 2009; Liu et al. 2013; Mackay et al. 2006; Schefold et al. 2009). They stabilise cerebral blood flow, improve ischemia-injured blood–brain barrier integrity and stabilise cell membranes in brain tissue and reduce glial damage (Ding et al. 2009; Heyes et al. 1992; Schefold et al. 2009). Additionally, SEV inhibits *N*-methyl-d-aspartate receptors and nicotinic acetylcholine receptors in a dose-dependent manner (Brosnan and Thiesen 2012; Ding et al. 2009). KYNA inhibits the same receptors (Bender 1982; Némath et al. 2005; Stone et al. 2003). Inhalation of SEV or isoflurane reduces perioperative brain injury (Dabrowski et al. 2010, 2012). Plasma KYNA concentration correlates with postoperative neuropsychological deficits in cardiac surgery patients (Forrest et al. 2011). An increase in plasma KYNA observed in patients with cerebral ischemia correlates with infarct volume (Brouns et al. 2010; Darlington et al. 2007). Volatile anaesthetics improve postoperative outcome in cardiac surgery patients (Kanbak et al. 2007; Rörtgen et al. 2010).

In summary, this is the first study documenting an increase of plasma KYNA in CPB patients and the effect of anaesthesia on KYNA. An increase in plasma KYNA correlates with severity of inflammation as measured by NLR. Our results suggest that SEV prevents the elevation of KYNA, most likely due to its anti-inflammatory properties. Based on our findings, KYNA can be used as a marker that directly correlates with the severity of inflammation in cardiac surgery patients.
